# Ethical Issues in International Research in Pediatric Psychology: Challenges and Opportunities

**DOI:** 10.1007/s10880-025-10087-z

**Published:** 2025-06-30

**Authors:** Rocío de la Vega, Eileen Chaves, Tatiana Lund, Gerald P. Koocher, Line Caes

**Affiliations:** 1 Department of Psychology, University of Málaga, Málaga, Spain; 2 Instituto Biomédico de Málaga-IBIMA Plataforma BIONAND, Málaga, Spain; 3 Department of Pediatrics, Nationwide Children’s Hospital, The Ohio State University, Columbus, OH USA; 4 Department of Psychology, University of Calgary, Calgary, Canada; 5 Department of Behavioral Sciences and Psychiatry at Boston Children’s Hospital, Harvard Medical School Bioethics Center, Boston, USA; 6 Division of Psychology, Faculty of Natural Sciences, University of Stirling, Stirling, UK; 7 Personalidad, Evaluación y Tratamiento Psicológico, Facultad de Psicología y Logopedia/Universidad de Málaga, C/ Doctor Ortíz Ramos, 29190 Málaga, Spain

**Keywords:** Ethics, Pediatric psychology, International research, Vulnerable populations, Adverse events

## Abstract

The rapidly expanding international scope of pediatric psychology presents significant collaborative opportunities as well as ethical challenges. In a quest of common ethics terminology and primary ethical considerations across international borders, we conducted an online survey with open-ended questions focusing on procedures essential to obtaining ethical approval for research with pediatric patients. Participants from 14 countries responded. This report provides an overview of some key international differences and challenges while providing recommendations for addressing each aspect. Key factors include ensuring international collaborators begin ethical planning from inception of the project; identifying pediatric ethics thought and policy leaders in each country; and confirming pertinent policies and procedures in each location.

## Introduction

Those interested in pediatric psychology have doubtlessly read the work of colleagues from other countries and have interesting ideas for research on topics that reach across national boundaries. Pediatric psychology research inherently involves interdisciplinarity and requires collaborations across different health care professions such as psychology, medicine, nursing, and occupational therapy, to name a few (Gance-Cleveland & Ozkaynak, 2021). Research involving children and families also raises additional issues of obtaining consent, permission, and assent that do not apply when all research participants are competent adults. Considering the structure of ethical research standards and approval requirements for research with pediatric patients creates a particularly complexstarting point necessitating thoughtful international collaborations.

The international character of pediatric psychology research has grown significantly, as demonstrated in the increasing international membership of the Society of Pediatric Psychology (SPP). At the end of 2023, SPP had more than 140 international members and national pediatric psychology organizations have begun to develop across the world (Simons, 2018). The European Paediatric Psychology Network (EPPN) evolved as a section of SPP in 2020. This growth has further stimulated an appetite for international research collaborations in pediatric psychology, as illustrated in a special issue of *Clinical Practice in Pediatric Psychology* addressing the “Global Reach of Pediatric Psychology” (Simons, 2018).

Despite the importance and growing prevalence of international pediatric psychology research projects, many challenges and regulatory barriers exist. One of the most significant stumbling blocks for such international projects involves acquiring ethical approval and participant consent for pediatric research. For instance, obtaining such participant consent requires a complex interaction among the researchers, participants (i.e., children), and their legal guardians in order to conduct meaningful research in an appropriate, safe, and legally compliant manner (US Food & Drug Administration, 2022). Complexity is further added for international projects (i.e., when researchers conduct research across different countries or settings, including settings that are different from their usual setting), as it is highly likely that legal requirements and societal expectations for assent, consent, permission, and participant safety will vary widely between participating nations and type of setting. Finding a way to anticipate and address such differences in approval requirements and processes at early stages of project development could significantly smoothen and expedite project approval.

Furthermore, given the growth in multiculturality within societies, it has become increasingly important within pediatric psychology practice and research to become aware of, respect, and adequately incorporate relevant ethnic and cultural differences. This need for increased understanding of such differences further underscores the relevance and need for international projects, as these may complement and offer new perspectives on pediatric patients’ needs and backgrounds. Such international alliances can help to generate more generalizable research and clinical utility. One potential consequence involves the need to modify study procedures (e.g., participant recruitment, formulation or translation of protocols, or data collection and storage strategies) to align with ethnic and cultural expectations (e.g., understanding differences in how health care systems, economic factors, and educational terminology cross-calibrate for international comparison) (Villagran, 2022). Fortunately, some frameworks for cultural adaptations of psychological interventions do exist (e.g., Barrera & Castro, 2006; Heim & Kohrt, 2019; Perera et al., 2020) and offer guidelines in this process. Adjusting practices for cultural sensitivity aligns with SPP’s mission to promote diversity and inclusion in pediatric psychology research and clinical care goals as well as their multicultural guidelines (see: APA Task Force on Re-envisioning the Multicultural Guidelines for the 21st Century, 2017).

In sum, a strong imperative exists for pediatric psychologists interested in international projects to more easily assess, understand, and apply differences that regulate research across international boundaries. Understanding these similarities and differences aids in anticipating ways of expediting the ethical approval process for pediatric research with children across national borders. This paper aims to describe a common language of ethics terminology and summarize the main ethical considerations in international research projects with children, with relevant examples across several countries on all continents. Readers will find a list of key definitions in Appendix 1.

## Status of International Ethics Regulations in Pediatric Psychology

In order to create an overview and summary of the current status of ethics regulations and procedures around the world, members of the International Committee of SPP designed an online survey covering the main elements of ethical practice that come into play when conducting pediatric psychology research. Using existing international networks, such as the Society of Pediatric Psychology International Committee, the survey was distributed to research collaborators located in various countries covering all continents, to capture real-world challenges and differences pediatric psychologists encounter when seeking ethical approval for research.

Potential respondents were sent an e-mailed invitation explaining the purpose of the survey and provided a link to access the data collection tool. Participants indicated their consent and confirmed agreement to share their data via publication by clicking on the survey link. One participant with research experience, including having been responsible for submitting ethics applications, per country was considered sufficient to provide descriptive information on national practices. Participation was voluntary and uncompensated. The study was considered exempt from IRB review by the first author’s institution experts as it represents a professional audit, using a survey to gather information from a professional audience with anonymous data reporting. All respondents were informed of this approach. The survey did not seek any personal or sensitive information, and participants did not include patients, minors, or vulnerable populations.

In addition to questions identifying the participant’s country and experience with obtaining ethical approval for research, the survey contained 10 open-ended questions allowing the participant to provide details on the various procedures followed to obtain ethical approval for research with children in their country (e.g., recruitment; consent, assent, and permission; safeguarding privacy; data storage; debriefing; language; and study design considerations). We aimed to include participants with a wide range of experiences of applying for ethical approval to be able to provide a comprehensive picture of the relevant information about the requirements and ethics authorities in their country. Data collection took place between November 2021 and September 2022. A copy of the survey appears in Appendix 2.

The survey obtained information from 14 pediatric psychology researchers based in 14 different countries working on different types of institutions: pediatric hospitals, Universities, and Research Centers. Table 1 summarizes the responses by country and ethical topic. Below, we summarize relevant issues that emerged from the survey responses, to provide a comprehensive overview of the responses to the survey questions, based on the most important ethical considerations worldwide, and illustrate potential differences that occur. For each of the ethical considerations, we provide recommendations based on existing literature and the authors’ own international experience. We did not apply any formal qualitative analyses technique (e.g., thematic or framework or content analyses), as this was deemed inappropriate for our purpose of providing a comprehensive, narrative overview of the different ethical process considerations across the included countries. Consequently, no summarization took place, and all of the survey data were organized following the different phases of study preparation and ethics clearance. All the information was included in the article, with only redundant examples being omitted.

**Table 1:** Survey results organized by country and ethical procedures required for each topic when conducting research with minors

	Consent and assent	Data storage and protection	Safeguarding procedures	Recruitment	Debriefing	Age-appropriate language
Australia	16 years old (14–16 is a gray area)	Differs by institution. Generally, password protected (or locked filing cabinets), backed up onto institution's network storage, de-identified where possible. For studies involving youth (< 18), according to the National Standards, data are kept until 7 years after the publication of the research or until the child turns 25 (whichever is later), then destroyed	Different states in Australia have different exact safeguarding In New South Wales all people working with youth need to have a "Working With Children Check". It involves a National Police Check (criminal history record check) and a review of reportable workplace misconduct Institutes have their own in-house ethics training regarding responsible research practices	–	Debrief: Parent- and child-appropriate debriefs are provided either written or verbal, as required by the research studies Adverse events: Any adverse events that arise due to research are reported by the research team to the chief investigator as soon as the research team learn of them. All adverse events are documented, and serious adverse events are reported to the Institutions (always within 72 h)	Differs between institutes. Generally, there is required to be both parent and young-person information sheets, the latter with age-appropriate language and concepts. Young people should be asked to summarize the information sheet. Verbal/written assent is project dependent. Debriefs are provided if relevant to the project and age-appropriate summaries of overall, amalgamated results are provided if requested
Canada	No legal age It is decided by the ability to provide informed consent, (as young as 12–14 years old)	Institutional standards of at least 5 years post publication for non-interventional research	The Tri-Council Policy Statement: Ethical Conduct for Research Involving Humans (TCPS) 2 certification required; Research team CVs are kept on file by our REB; at our institution anyone who is in a position where they may be alone with a minor must have a vulnerable sector search Criminal Record Checks required by our institution upon hire for all positions	Study dependent, must be explained and approved by the institutional ethic board School recruitment usually need additional approval by the local school board	Required; study dependent; standard wording created by the institutional REB is included on consent forms with information on how to contact both the PI and the REB with questions or concerns regarding participation	Study dependent; Institution dependent; consent and assent forms are assessed for language level and clarity during the approval process Young people should be asked to summarize the information sheet
Denmark	15 years old and above Parents should be informed if they are minors	GDPR should be followed; data are usually kept for 5–10 years	We regularly need to complete training regarding ethics and or safety for data storage	For schools, the school director needs to approve For clinics, the specific clinics need to approve	I am not aware of this	The information about the study is usually provided in paper or on an electronic form. Information about being able to withdraw from the study at any time are usually presented in the informed consent
Hong Kong	18 years old	Anonymized data for analysis, data in locked room, password-protected computer. No other special requirements	No special vetting of research assistants	For schools, the school director needs to approve For clinics, the specific clinics need to approve	Mainly PI or contact person for PI	Paper information sheet. A copy to take home. Lay language Questions asked during consent process and contact number for further questions
Ireland	18 years old	Consent forms with personal details are typically stored separately from the dataset and the key linking the two would be password protected and only accessible by the research team. GDPR requires that data are stored for 7 years	Garda vetting (police vetting for working with minors). No other training requirements	Recruitment in the school sector is on a school-by-school basis, there is no ethics committee for the Department of Education Individual hospitals also have their own ethics committees and application forms	If issues arise relating to child protection, we would need to follow the child protection guidelines of the organization in which the research takes place. A key contact person may also be in place in individual settings to provide follow-up psychosocial support to participants (if required)	There are no national rules or guidelines in place for this. But best practice is to communicate in a developmentally appropriate manner with potential participants about who you are, what is involved; how children can decide if they want to take part and change their mind; ask questions, etc.
Kenya	16 years old If the research offers direct benefit not available otherwise, it falls under the scope of parental authority, overriding a child’s desires	Clinical trials: 15 years Other studies: 5 years	Ethics training is required	The procedures specified in the ethics application should be followed	If an adverse event occurs, ethics board and the study sponsor should be informed within 2 days	-
Malawi	Assents when they can understand what they can read. Consent > 18 years old	Data should be anonymized and kept confidential. Institutions are expected to establish research data banks and repositories. The requirements do not specify how long data should be kept	–	–	The proposal should have an explanation of whom to contact in the event of a research related injury	Consent form or information sheet, should not include intimidatory, threatening, or deceptive language. The consent should be in a language that the child or the parent can easily understand
Netherlands	12—16 years old: children and parents. Below 12: both parents sign	GDPR applies in the Netherlands. Storage of medical anonymized data may be up to 15 years after the last publication. Storage of non-anonymized data must be done separately (10 years)	Academic researcher should get a BROK certificate: https://nfu-ebrok.nl/?lang=en	Before recruiting, the study has to be reviewed by an accredited ethics board. When consent has been given, recruitment can start This is not applicable to population screening (special license is needed)	https://www.ccmo.nl/onderzoekers/publicaties/publicaties/2017/06/19/flowchart-ongewenste-voorvallen-geneesmiddelenonderzoek	Subject Information Sheets should contain age-appropriate language For children, a separate sheet is available
New Zealand	16 years old	10 years	Good practice certification is new in NZ, but completed by some researchers	Locality approval is required Indirect (non-coerced recruitment) is practiced Written consent is necessary before enrollment	For some studies, risk management processes with existing health practitioners are required. For higher risk studies, active oversight by data management committees are needed	All documentation must be provided and approved by the ethics committee. Flesch reading scores also need to be calculated for child-related sheets/forms
Portugal	18 years old or older. Parents sign if they are minors	Data are always coded in an anonymized manner	Researchers are encouraged to take part in courses concerning ethical aspects	Recruitment includes mainly the general population, but also the clinical population although sometimes access to it is much harder	–	Young participants invited to participate in a study are informed that their participation is voluntary and data will be kept anonymous and confidential
Spain	16 years old	An anonymized dataset must be warranted and data storage will last up to 5 years after data recollection	–	Only anonymized datasets can be shared among clinical centers and locations within a multicenter study	All adverse events have to be registered during the implementation of the study	The study protocol must be explained to child participants using age-appropriate language and researchers must let them ask any questions about the project
Sweden	15 years old, given that they are deemed to be capable	All data storage as well as GDPR needs to be in place and described in the information provided to participants. This letter is a required attachment to the ethics application	No such required as exemplified but safeguarding can be required in research studies in the form of procedures to prevent harm or problem escalation in study participants	Studies have to be described on a general level although some go into detail with specifics	Yes, needs to be described, a bit dependent on the sensitivity of the study subject	Yes, a central part of the ethics application
United Kingdom	16 years old	10 years at university	DBS usually but depends if via NHS or other sources that require this	If clinical, then likely the healthcare practitioner recruits	Yes, various support organizations	-
United States	12 years old	Separate data and identifiers	HIPAA training Institutional accreditation	Each institution needs to have their own institutional review board approval	This varies by institution and the study	It is recommended that study materials are written/prepared at a reading level that is accessible to most study participants

## Ethics Authorities and National Rules

When planning a study, researchers should initially determine if the country targeted for the study has a national ethics authority. Reviewing any such national standards for their study will prove essential. Please see Appendix 3 for a comprehensive list of national authorities by country, of the countries represented in the survey. We found that whereas some countries have a nationwide ethics system for research with human participants and a national authority (e.g., Canada, Kenya, New Zealand, UK), not all countries have standardized ethical guidelines or a single national authority. In these cases, regional or local authorities or even institution-specific authorities determine policies (e.g., Ireland, Portugal, Spain).

However, even when a national ethics authority is in place, the responses identified significant individual institutional differences that also demand consideration beyond the differences found between countries. For example, one respondent indicated that their institution differentiates between intervention (e.g., randomized clinical trials) and non-intervention research (e.g., qualitative or survey research). If the research does not include intervention, the research project does not require review by and approval by a national ethics board, but only by the institutional authority (see Table 1 for details). As another example, in the US institutional quality assurance or quality improvement research is considered exempt (US Department of Health and Human Services, n.d.). Such research includes activities whose purposes are limited to improving the quality of patient care or collecting data regarding implementing clinical, practical, or administrative goals. Consequently, even in countries with a national ethics authority, researchers need to also familiarize themselves with specific institutional ethical requirements.

### Recommendation: Establishing an Ethics Lead for Each National Team in the Study

One strategy for overcoming this (and many other potential barriers described below) involves designating an ethics lead person for the project within each country involved. The national ethics lead will play a role in contributing to the ethical approval planning of the project from its inception. This role may sometimes fall to the principal investigator, an experienced research coordinator, or a local collaborator, as appropriate for the study. This national ethics lead should confirm local policy and procedures with their own institution, and engage in conversations with their local ethics boards. In many institutions, ethics boards (i.e., institutional review boards, IRBs, in the United States) can provide consultation when planning a study, to get advice on “gray areas” or sensitive topics. Working together as a multi-national team, the various national ethics leads can create a summary of ethical requirements by country. Ensuring that every aspect of the research process complies with the requirements of the country and institution with the most rigorous rules and regulations is key to a project’s success.

## Variable Consent and Assent Policies

The ages allowed to give consent or required for assent vary widely by country, ranging from 12 to 18 years old, with some countries requiring parents to consent at all ages if participants are below the age of legal majority. For example, in Canada the age at which a child can consent without also obtaining parental permission varies on a case-by-case basis, depending on the nature of the study and the characteristics of the participant. On the other hand, the approach in Ireland is conservative in that 17-year-old participants still require parental permission for all types of research. In some countries, such as Kenya, if the research offers a potential benefit that is not available outside of the research setting, parents or legal guardians have the power to grant consent, and child assent is not needed. This practice might lead to special therapeutic misconception problems; for instance, if the study is a randomized clinical trial where potential benefit exists in some study conditions, investigators must ensure that those giving consent understand they may not be assigned to a treatment arm of the study and thus may not get any benefit from the treatment before consenting. For more details on how to proceed, we advise readers to consult the provided links in “Appendix 3.”

### Recommendation: Adapted Consent Documents

Recognize from the outset that different consent or participant information documents may be needed for each country involved in the study. The national ethics leader will need to assess whether the language, tone, and content of the documents participants will receive align culturally and legally to requirements and expectations. Any language translation should undergo back-translation (e.g., translate from language A to language B, and then from B to A) to adequately capture the original meaning. Usage review may be required even when two countries share the same language. For instance, English speakers in Britain use the term “scheme” to mean plan or project (e.g., highway construction scheme or organ donor scheme). But in American English, a “scheme” connotes a devious plot (e.g., a money-laundering scheme).

## Data Storage and Protection

Significant differences in data storage requirements exist across national boundaries. In most countries, consent documentation and anonymized data are stored in different locations. The preservation duration for research records and data varies from 5 to 15 years and often depends on the institutional policies rather than law (see Table 1 for details).

Data protection requirements also vary by country location. European Union countries are subject to the General Data Protection Regulation (GDPR) (European Commission, 2016), while health-related data in the United States are subject to regulations promulgated under the Health Insurance Portability and Accountability Act (HIPAA) (US Department of Health & Human Services, 1996).

### Recommendation: Data Management Plan

Create a data management plan (DMP) to satisfy the most restrictive country’s or institution’s requirements and comply with all the applicable data protection laws. There are freely available websites that walk researchers through the development of the data management plan (DMP). We recommend consulting the guidance page of “DMP Online” (Digital Curation Centre at the California Digital Library, n.d.) which provides examples of plans that comply with the requirements of international funding bodies such as: the UK Research Council, the US National Institutes of Health (NIH) or the HORIZON Europe funding scheme. Please see Table 2 below for more information.

**Table 2:** Data management plan

Components of data management plans	Questions to consider
Data Collection	What data are being collected? How will the data be collected?
Documentation and metadata	What documentation and metadata—information describing the data—will be collected? How will anonymization and participant identification coding occur?
Ethics and legal compliance	Who will manage ethical and legal issues and how?
Intellectual property rights	How will authorship, copyright, or patenting of inventions be managed?
Storage and backup	How will the data be stored and backed up during the research? How will access and security be managed?
Selection and preservation	Which data are of long-term value and should be retained, shared, and/or preserved? What is the long-term preservation plan for the dataset?
Data sharing	How will the data be shared? What restrictions will apply to data sharing?
Responsibilities and resources	Who retains responsibility for data management? What resources are required to manage the plans?

## Safeguarding Procedures

The most common safeguarding procedures reported by respondents include requiring passing ethics and human participant protection courses or other vetting for research personnel prior to starting the research. For example, in Australia, Ireland, the UK, some US states, and Spain, researchers undergo criminal or child abuse background checks from police or public safety authorities to work with minors. In some countries, researchers must complete specific training. For instance, in the United States, researchers must complete HIPAA and Collaborative Institutional Training Initiative (CITI) Good Clinical Practice Training before starting research (National Institutes of Health, 2022). Additionally, survey respondents indicated that institutions usually have additional ethics requirements, such as completing specific ethics training or reporting specific adverse events to their ethics board.

### Recommendation: Ethics Leaders

Each study’s national ethics leader should exercise responsibility to monitor country and institution-specific ethics training requirements, ensuring all study team members have completed respective country-specific requests, and confirming relevant trainings for all researchers involved. When creating the budget for the project, expenses for these training requirements and background checks should be considered.

## Ethical Aspects of Participant Recruitment

Differences between the standard processes to be followed for suitable recruitment varied at the institutional or site level rather than being related to the type of setting (i.e., clinical, research, vs community settings), according to survey respondents. Key differences or challenges reported concerned the authority of the recruiters and compensation offered to ensure that participation is not coercive.

### Recommendation: Start with the Most Strenuous Requirements for all Countries/Sites Involved

Looking at the most restrictive of the proposed sites and following a participant recruitment procedure adequate for that site will facilitate a unified procedure for all sites involved. National ethics leaders for the project have the responsibility for ensuring recruitment procedures are not coercive for potential participants. For example, in many regions, such as Asia or Latin America, healthcare providers are perceived as authority figures in control of a child’s medical care (Napier et al., 2017). This power imbalance when inviting participants may cause some participants/families to agree to participate in research to avoid disrespect or lose access to the provider’s care. Similar considerations may involve offers of compensation for participation. For example, offering a family $100 (US) to participate in a research project requiring multiple blood draws and an hour of time would equate to approximately 14,750 Kenyan Shillings. With two thirds of Kenyans living in poverty and making less than $3.20 per day (US Agency for International Development, n.d.), the equivalent of $100 US would be far more economically coercive in Kenya. Some might argue that the compensation offered to Kenyan participants should be lowered considering the average hourly wage in the US is approximately $28.34 (in 2023), and the average Kenyan would need to work more than 8 days to match the US hourly rate. Alternatively, we must ask whether social justice demands paying equivalent currency value for the discomfort and time or whether the national wealth imbalance would prove unfairly coercive. These are not simple matters, but they demand thorough ethical analysis and consideration with local collaborators and residents.

## Conducting Appropriate Debriefing and Reporting

Participants must have a way to monitor and report adverse incidents or harms that occur as the result of participating in research. They also may have an interest in knowing the findings of a study once it is completed. For these purposes, participants should have contact information of the person responsible for the study, a means to report adverse events that is free of charge (e.g., toll free number and email address), and the identity of an oversight bodies to be notified. Additionally, instructions on this process should be made available to participants in an accessible language. Such events must be captured and relayed to the ethical authority overseeing the study and to the institution’s ethics authority within a specified timeframe, to ensure timely identification of systematic risks. Also, participants must have an assurance of continued care, should they choose to withdraw from participating in a study at a facility where they normally receive care. If participation in the research causes harm to participants, remediation may be required. Those requirements generally apply across countries, differing only slightly as a function of specific institutional rules. For example, as reported by survey participants, some institutions require researchers to provide feedback on clinical assessments done for research purposes (e.g., if scores on a depression questionnaire reach a clinically significant level or suggest suicide risk, the researcher has a responsibility to provide that information to the participant and/or parent). Other institutions may not require sharing of primary or incidental findings, and some may even forbid informing about individual outcomes.

### Recommendation: Agree On data Collection Obligations and Make Plans

Create a comprehensive document, across all the national research groups listing the types of information each country and institution requires to be provided to the participant(s) as part of the consent process. Prepare a protocol for sharing this information with participant(s) in a culturally appropriate manner. Specify how primary symptoms and incidental findings will be handled. If the countries involved differ with respect to what information must be shared with participants, researchers should coordinate with the local regulatory boards of each country and attempt to find a solution that affords fair treatment to all participants. For example, the correct language needed to communicate clinically significant symptoms should be calibrated to avoid embarrassment in countries with high levels of mental health stigma.

## Use of Developmentally Appropriate Language

Use of developmentally appropriate language becomes critically important when discussing key research concepts such as the right to voluntary withdraw from a study at any time, logistical details about how to provide information about the study (e.g., verbal vs. written, parents or caregivers present or absent to avoid coercion), and how to gauge participants’ understanding of their rights. Details regarding preparation and sharing such information typically vary by institution, with some country-specific requirements. For example, in New Zealand, the Flesch Reading Score must be calculated and reported. In general, age and developmentally appropriate language should be used that corresponds to the age and developmental level of minors in the study and the reading level of parents. Many IRBs in the US suggest an 8th grade reading level target.To gauge understanding of the study information, child participants can be asked to summarize or repeat back the content of the explanations given or to answer questions about their rights as a participant and about the study procedures.

### Recommendation: Adapt Consent and Other Study Information to Participant Reading Levels

Adaptations to the documentation provided to participants, including debriefing procedures and scripts, require not only language translation but also need to be age- and culturally appropriate. As stated before, the ethics leader in each participating country should oversee use of language pegged at an appropriate developmental level, cultural and language context (with the collaboration of bilingual/bicultural colleagues or professional translators, as needed). The local ethics leader should also make sure any debriefing scripts and procedures adhere to country-specific and institutional requirements. When creating the budget for the project, the cost of any language and translation service expenses should be considered.

## Assessing and Reporting Adverse Events

Rules related to the types of reportable adverse events (e.g., injuries or life-threatening events), the timeframe for reporting (e.g., within two weeks) and the authority designated to receive reports depends on the institutions involved, funding agencies, and government regulations. For example, in Kenya, if an adverse event occurs, the local ethics board and the study sponsor should be informed within two days. In Australia, the timeframe allowed is three days.

### Recommendation: Understand Who Must Receive Adverse Event Reports And reporting Deadlines

Investigators must understand who has responsibility for reporting adverse events, what details must be included, and who must receive such reports (i.e., IRBs, ethics boards, study sponsors, etc.) and by when (i.e., deadline or timelines). Creating a decision tree for the study (see, for example, Appendix 4) and making it available to all study members can help to ensure meeting the requirements.

## Special Circumstances

In many countries surveyed, researchers report that all children are classified as vulnerable research participants by their country’s and institution’s ethics boards. However, even within the larger category of ‘children,’ researchers should highlight the importance of ethical considerations for *special and vulnerable subsets* of children and adolescents, such as non-verbal participants, participants with chronic illnesses, disadvantaged minorities (including sexual minorities or gender expansive youth), and participants with cognitive deficits. For example, in the Netherlands, scientific research with participants younger than 16 years old is prohibited unless: 1) the research is of direct therapeutic benefit to the research participant(s), or 2) that type of research can only be conducted with participants younger than 16 years old (e.g., validating a questionnaire specific to minors). In this case, the risks and burdens of the research must qualify as minimal in comparison to the standard treatments given to individuals in similar circumstances (e.g., other children of similar age with the same medical condition). If no standard treatments exist, the nature and severity of the potential participants’ medical condition is considered. In Portugal, certain groups of young people in under-studied categories, such as deaf individuals, require special consideration by ethics boards as minorities that may require incorporating additional supports or protections. Ethical data collection should respect the limitations of individuals in each special case and researchers should adapt the format and structure of their interventions accordingly. If potential participants’ vulnerabilities cannot be accommodated within the methodology of the research protocol it may prove necessary to exclude them from the study. In Malawi, special considerations apply to orphans or children with HIV. Researchers working with such vulnerable persons are required to implement extra protections or safeguards for their safety and welfare (e.g., to protect privacy and minimize stigma).

Additional issues were identified when conducting international research, including research largely shifting from face-to-face interactions to an online, digital format during the COVID-19 pandemic. Many researchers had to learn how to conduct virtual assent and consent procedures, use secure online video-conferencing platforms to conduct interviews, and create digital interventions. Understanding ethics boards’ requirements for digital interventions is also important and often varies by country. For example, in Denmark, a minor can use a smartphone app, computer, or website unsupervised at age 15. In Portugal, however, researchers are required to monitor the use of digital interventions (i.e., apps), diaries (i.e., symptom diaries), and clinical homework given to participants outside of study sessions (i.e., home-based practice of an intervention) for adverse events. In this case, if such data were managed by a researcher located in a different country, that person should contact the ethics leader in Portugal for guidance on handling that data in compliance with national rules. Many participants noted that their institution’s ethics board has approved the use of a digital consent processes, including digitally signing consent and assent forms (e.g., the REDCap e-consent framework as described by Lawrence et al., 2020).

Finally, in the case of research with participants who speak different languages, written study materials should be translated and back translated from the language of origin by qualified individuals to avoid an inappropriate change of meaning or a reading level beyond the ability of the target population. This may involve translation by a bilingual psychologist or by a professional translation service and then a reverse translation check by a different psychologist who is a native speaker of the initial language of the instrument or study documents.

An additional aspect to consider involves incremental costs or expenses related to specific ethics requirements. For example, in Sweden and Malawi ethics boards charge a fee for conducting reviews. In Spain extra fees apply only if the researchers are affiliated with private institutions. Recognizing when additional expenses apply and including them in the budget will be essential.

## Illustrative Examples

As noted earlier, the extant literature on ethical considerations for international projects involving minors is sparse. However, there are some notable exceptions of published studies that illustrate how to approach the ethics process in international studies. One example involves a protocol to study the effects of the COVID-19 pandemic on mental health outcomes in children and their caregivers (de Young et al., 2021). The authors explain how the main research site had responsibility for providing standardized study materials (including ethics forms) to lead researchers in each country and how each national team on the project retained responsibility for their part of the research. Country leads at each site reported back to the main site after ethical approval was obtained. Another example involved obtaining pediatric assent in an international study using antiretroviral therapy (Vreeman et al., 2009). The authors reviewed the ethics process and summarized the contact with ethics boards in the United States and Kenya. They gave descriptions of some points where requirements were not clear and challenges occurred, offering suggestions on how to overcome such international issues in future. Finally, a review article focused on consent in pediatric research in low- and middle-income countries (Colom & Rohloff, 2018) and included a summary of ethical considerations found in several articles with illustrative cases of research conducted in different countries.

## Limitations

The findings of this study need to be considered with caution, as only one respondent per country was included. Despite all of them having previous experience with ethics applications and being knowledgeable of ethics authorities and standards, some institutional differences may have led to their specific responses. In that sense, drawing conclusions about setting-specific differences should be done with caution, given the study design.

## Conclusions and Future Directions

This paper summarizes and reports on first-hand perspectives of challenges in ethics procedures when conducting research across national boundaries. Colleagues from 14 countries described key national differences and made recommendations on overcoming potential challenges. Although the results are limited by having only one respondent for each of the represented countries, key insights were collected. Heterogeneous mandates and rules apply internationally and even between institutions within a single country. Based on information retrieved in the survey, we provide a set of recommendations for use in ethics planning of international studies involving minors.

Specifically, we recommend the following: (1) ensuring that national collaborators knowledgeable about research ethics practices from each country have a role in the planning from the inception of the project; (2) designating a colleague as the ethics leader for the study per country; and (3) confirming all pertinent local regulations, policies, and procedures with each site. Periodic coordination meetings between the project’s ethics leaders would also ensure that a systematic approach is followed and would provide a support network to discuss any ethics issue that may emerge or any change on national or institution requirements during the development of the project.

Study authors should consider comprehensive and transparent reporting of ethical issues and actions taken in any resulting publications. For example, including supplementary materials on the ethics process of a study when conducting international studies with minors would help future investigators when planning appropriate and optimal ethics processes. Similarly, we would encourage the publication of informative case studies that parse how ethical issues in international settings were addressed, as they can be helpful in illustrating practical ethical challenges and fostering nuanced understanding that may not emerge from broader methodological approaches. Such efforts will ultimately facilitate much needed international collaborations, studies with multicultural and multiethnic populations, and the standardization of international ethics protocols.

## Data Availability

Data available within the article, tables, and appendices.

## Appendix 1: Key Definitions and Cultural Reframing

Understanding fundamental ethical concepts and how these vary in interpretation and priority across legal systems and national boundaries will prove essential when considering international ethical clearances for pediatric research. The definitions typically flow from Western cultural values, but also require information from other traditions to grasp complexities of international research ethics. We must apply these terms and values with caution. For example, the culturally established ethical norms in a patriarchal society become a way to maintain the inequality of women. Or the cultural norms in a caste-based society may justify discrimination against members of the lowest caste or trivialization of their rights.

Consider the concept of Ubuntu as reflected in the African perspective. In essence, the community takes precedence over the individual and community well-being takes priority over individual inalienable rights including autonomy. This indigenous concept rooted in culture and religion forms a key element of collective consciousness in many African nations (Chuwa, 2014; Roux & Coetzee, 2001). Ubuntu ethics conceptualizes fundamental human rights in the context of communal rights, hence, understanding human rights requires focusing on the context of the society in which the individual lives.

Pressing for the involvement of community in any individual’s consent process presents the risk of breaching fundamental human rights, especially those of women, children, elders, or other vulnerable people who have limited power of autonomy. There is a possibility that these vulnerable people could face coercion under the pressure of communal norms dictated by dominant community leaders or heads of families, who are typically male. Similarly, according to Ubuntu philosophy, disclosing to a patient how advanced their cancer has progressed, instead of telling their family, would qualify as offensive and rude behavior that might adversely affect the patient’s willingness to continue in treatment (Ekmekci & Arda, 2017). In Western culture, providing such detail shows respect for patient autonomy. Asking adherents of Ubuntu for informed consent to participate in clinical trials without first informing other members of the community in advance would qualify as unethical. In Western culture, discussing such matters with the family of a patient in absence of their approval would constitute a violation of privacy and confidentiality (Ekmekci & Arda, 2017). Consequently, the definitions below offer a general starting point and require consideration within their local context and culture.

*Consent* (sometimes called “informed consent”) refers to the process by which a participant voluntarily agrees to participate in a research project based on information they can readily understand regarding all aspects of the project that might reasonably influence their willingness to participate. Although written documentation is typically required, obtaining consent is best conceptualized as a process. In some countries, especially when literacy is low, political fears abound, or signing one’s name to documents is intimidating participants may resist written consent forms (Aguila et al., 2016; Bolado et al., 2024). In North America, Europe, and Australia consent can only be given for oneself, must always be fully informed, and may be revoked (Koocher & Keith-Spiegel, 2016; Manti & Licari, 2018). In nations or cultures where community engagement expectations, as with Ubuntu, come into play, more elaborate processes may be required.

*Assent* is the term used to express willingness to participate in research by persons under the legal age of consent or incapable of expressing themselves. Western concepts of autonomy require that such individuals must have access to information on the proposed research, but also have the right to veto participation in many situations. Veto authority typically applies when no direct benefit or health need exists. If assent is given, informed consent must still be obtained from the person’s parents or guardian (US Department of Health and Human Services, 2024; Cayouette et al., 2022; Koocher & Keith-Spiegel, 2016).

*Permission* refers to the process by which a person below the legal age or otherwise unable to give consent to participate is voluntarily enrolled in a study by a parent or legal guardian based on full information that they understand and agree to participate (Koocher & Keith-Spiegel, 2016; US Department of Health and Human Services, 2024). In some cultures, permission-seeking must also include societal leaders.

*Review Panel*s refer to governmental bodies or quasi-governmental groups (e.g., IRBs or Institutional Review Boards, mandated by law in the United States but not run by the government) that review and approve research protocols and consent procedures. Typically, these are groups of trained professionals and lay people who work together to ensure that all research conducted at an institution upholds the principles of medical ethics and ensure that safety protocols are followed and participants are protected from undue risks (Amdur & Bankert, 2011).

*Safety and adverse events* refer to the fundamental principle of protecting research participants from physical, psychological, social, or material harms and preserving the health and well-being of individuals and the community (Québec WHO Collaborating Centre for Safety Promotion and Injury Prevention et al., 1998). Adverse events range from mild to severe (including death) and may result from any aspect of a research or medical protocol or by random events occurring during the period under study (de la Vega & Palermo, 2021).

## Appendix 2: Survey on Ethical Procedures for Pediatric Psychology Across Countries

The aim of this survey is to better understand the general differences on ethics procedures for pediatric psychology studies across countries in a range of topics. Please answer the questions below focusing on the Country you work at. Be as detailed as you find necessary. You can skip any question you do not have knowledge about.

### General questions

- Which country are you currently working in (and basing your responses on)?
- Is there a national ethics authority to retrieve updated information or guidelines from? (if so, please provide the name and website)
- Have you cleared ethics for studies in that country before?
- Could we contact you in case we have additional questions or clarifications? (if so, please provide your name and email)

### Open-ended questions

- What are the main ethical aspects to consider before starting a study in children or minors in your country?
- What are the procedures that need to be in place with respect to …
- *Consent and assent procedures* (e.g., at what age can children provide consent for themselves)?
- *Data storage* (i.e., different storage of consent info and anonymized data, and how long data can be or should be kept after a study is done) and data protection requirements (e.g., GDPR in Europe and HIPAA regulations in the USA)
- *Safeguarding procedures*, including any courses/vetting or certificates the researchers need to complete/obtain (e.g., police background checks: general or sexual; ethics training)
- *Recruitment*, including recruitment across various clinical centers and locations (i.e., schools vs clinics vs community)
- *Debriefing*, including resources to be provided in case the child experiences distress due to taking part (i.e., who to report them to (e.g., ethics board, study sponsor), and by when (deadline)).
- *Demonstrating the use of developmentally appropriate language* on key research aspects (e.g., being able to withdraw at any time), including how to provide the information about the study (e.g., verbal vs on paper, with or without parents present to avoid parental coercion) & how to gauge their understanding of the rights of a research participant
- *Specific design-related considerations* (e.g., simple online survey vs randomized control trial). For example, age when a minor is allowed to use an app or website unsupervised.
- *Considerations for special cases*: vulnerable populations (non-verbal children, children with medical conditions or disabilities), digital health studies (e.g., how to consent online, how to monitor adverse events)
- Is there anything else you would like to add?

Thank you for your time and help!

## Appendix 3: National Ethics authority by country

**Table:**

Country	National authority	Website and details
Australia	Yes	NHMRC https://www.nhmrc.gov.au/about-us/publications/national-statement-ethical-conduct-human-research-2007-updated-2018
Canada	Yes	Tri-Council Policy Statement: Ethical Conduct for Research Involving Humans—TCPS 2 (2018) https://ethics.gc.ca/eng/policy-politique_tcps2-eptc2_2018.html
Denmark	Yes	Danish Ethics Committee https://en.nvk.dk
Hong Kong	No	-
Ireland	No	There are multiple individual ethics committees with different guidelines and procedures. The Department of Child & Youth Affairs, has some guidance for working with minors: https://www.lenus.ie/bitstream/handle/10147/311115/xEthicsGuidance.pdf?sequence=1&isAllowed=y
Kenia	Yes	https://www.kemri.go.ke
Malawi	Yes	National Committee on Research in Social Sciences and Humanities https://www.ncst.mw/national-committee-on-research-in-the-social-sciences-and-humanities/
Netherlands	Yes	https://www.ccmo.nl/
New Zealand	Yes	NZ HDEC https://ethics.health.govt.nz
Portugal	No	-
Spain	No	There are some regional and local (universities or hospitals) ethics authorities
Sweden	Yes	etikprovningsmyndigheten.se
UK	Yes	HRA if NHS related. BPS if broader psychology
USA	Yes	US Department of Health and Human Services Office for Human Research Protections (OHRP) https://www.hhs.gov/ohrp/index.html

## Appendix 4: Processes to Develop an Ethics Assessment for International Pediatric Psychology Projects: Considerations for Ethics Board Reports and Scientific Papers

In order to facilitate ethics applications for international projects, a step-by-step time-based guide will be presented to help researchers successfully consider and implement all the ethically relevant aspects of an international, pediatric psychology research project. The guide is partially adapted from the one included in a chapter about assessing and reporting adverse events (de la Vega & Palermo, 2021).

### Setting up the Project

1. Review the literature: look for international projects in similar countries to learn from other researchers’ approaches.
2. Access ethics authorities from the countries and institutions of interest.
3. Contact local colleagues and appoint an ethics leader in each country.
4. Contact the SPP International Committee for additional guidance or questions, as needed, or to help find a local colleague.

### Before the Ethics Application

Identify relevant ethical aspects based on important sample and trial (or treatment) characteristics. Some important things to consider:

1. Who is the population being studied?
  - Do they have special vulnerabilities?
  - Can they communicate the presence of adverse events?
  - Do participants need to give assent or consent? At what age?
  - Who can withdraw from the study? Can a child withdraw if they are the participant?
  - Which languages do participants speak?
2. What is the study design?
3. In case of an intervention study, what is the treatment or intervention being studied?
  - What are the known risks and usual adverse events (if any)?
  - Which specific procedures are going to be used and which adverse events can be expected from them?
4. In case of an intervention study, how is the intervention being delivered?
  - Is the intervention being delivered to an individual, a family, or a group?
  - Is the study virtual or are there face-to-face study visits?
5. What is the best method and timing of assessing adverse events?
  - How long is the treatment or study procedures? (e.g., 3 months, 8 sessions, 1 year)
  - Can the assessment points be used to include adverse event monitoring?
  - Will a psychologist see/interact with participants in-person?
    i. If so, what opportunities exist to add adverse event assessment?
    ii. If not, what alternatives can be put in place?
6. What measures are available to standardize the capture of adverse events?
  - Do any of the published measures fit with the study characteristics?
  - Is it necessary to develop a questionnaire ad hoc?
  - Is patient self-report or therapist report possible?
7. If not, can a family member or an observer report?
8. Can data be obtained from institutional records, such as clinical histories or school reports?
9. Are there costs associated with obtaining ethical approval?
  - Does the ethical board approval process come with a cost?
  - What are the costs associates with obtaining appropriate researcher certificates (e.g., background checks)?
10. What is the typical timeline to obtain ethical approval in each involved country?

### Preparing the Ethics Application

1. Set up privacy and safety safeguard procedures.
2. Ensure age and developmentally appropriate language of materials (as well culturally appropriate materials).
3. Create informed consent and assent forms (that have been translated, if needed, and culturally adapted to each country).
4. Set up procedures for assessment and reporting of adverse events.
5. Create debriefing protocols.
6. Create an ethics-compliant data management plan.
7. Train all the relevant team members on the ethical and international aspects of the study.

### During the Study

1. Follow local policies of the institution and regulatory agencies for safety monitoring (IRB, sponsor and government requirements). Report serious adverse events to regulatory agencies; these have a regulatory definition that includes death, life-threatening events (e.g., suicide attempt), hospitalization (e.g., medical or psychiatric), disability or permanent damage, birth defects, required intervention to prevent permanent impairment or damage, and other serious important medical events (e.g., seizures/convulsions).
2. Collect data on adverse events at each planned assessment point.
3. Follow up on participants who withdraw or drop out of the trial. Does the participant attribute withdrawal to:
  - The psychological treatment (e.g., specific treatment procedures)?
  - Other study procedures (e.g., assessment protocols, having to travel to receive the treatment)?
  - Non-related reasons (e.g., a car accident, unemployment)?
4. Maintain the created ethics-compliant data management plan

### After the Study has Finished

1. Report adverse events according to existing detailed reporting guidelines. Report details within each treatment group, including:
  - Nature of the adverse event (i.e., What happened?)
  - Frequency (i.e., How many times did it happen? How often?)
  - Severity and relatedness (i.e., Was it severe, moderate, or mild? Was it attributed to treatment?)
  - Timing (i.e., When did it happen? How long did it last?)
2. Include a clear description of the ethical procedures and findings in any publication derived from the study. As a general consideration, two main aspects should be addressed: transparency and reproducibility of the process. In other words, clearly reporting the procedure followed in a detailed manner would allow readers of the research to reproduce the ethics process followed in the study, contributing substantially to the development of ethically sound international projects.
3. Save and/destroy the data as per the created ethics-compliant data management plan.
